# Microstructure analysis of 8 μm electrolytic Cu foil in plane view using EBSD and TEM

**DOI:** 10.1186/s42649-022-00071-4

**Published:** 2022-03-28

**Authors:** Myeongjin Kim, Hyun Soon Park

**Affiliations:** Department of Materials Science and Engineering, Inha University, Incheon, 22212 Republic of Korea

**Keywords:** Electrolytic Cu foil, Sample preparation, Electron backscatter diffraction, Transmission electron microscopy

## Abstract

With the lightening of the mobile devices, thinning of electrolytic copper foil, which is mainly used as an anode collection of lithium secondary batteries, is needed. As the copper foil becomes ultrathin, mechanical properties such as deterioration of elongation rate and tear phenomenon are occurring, which is closely related to microstructure. However, there is a problem that it is not easy to prepare and observe specimens in the analysis of the microstructure of ultrathin copper foil. In this study, electron backscatter diffraction (EBSD) specimens were fabricated using only mechanical polishing to analyze the microstructure of 8 μm thick electrolytic copper foil in plane view. In addition, EBSD maps and transmission electron microscopy (TEM) images were compared and analyzed to find the optimal cleanup technique for properly correcting errors in EBSD maps.

## Introduction

Lithium secondary batteries have been widely used as batteries for smartphones and laptops as well as the power source for electric vehicles. (Park 2012; Pender et al. 2020) Materials mainly chosen as anode for lithium batteries include electrolytic copper foil and graphite. (Kepler et al. 1999; Obrovac and Checrier 2014) Electrolytic copper foil used in lithium-ion batteries has no restrictions on possible length and it is easy to produce thinly, while it also has poor mechanical strength and is prone to warping. (Ibanez and Fatas 2005) The thickness of copper foil has become thinner due to recent miniaturization of mobile devices, increasingly implemented ultra-fine circuits and increased energy density. (Woo 2016; Borah et al. 2020) The thickness of copper foils commonly used for electric vehicle batteries is about 4 to 15 μm. Many researches such as adding additives or heat treatment are being performed to improve the mechanical properties such as the deterioration of elongation and tearing easily due to ultra-thinning of copper foil. (Woo et al. 2013; Hatano et al. 2000; Nowell et al. 2005) In addition, their strength and ductility of foils generally reduce with a reduction in the thickness due to the size effect (Courtney 2000; Kim and Rhyim 2016).

In order to improve mechanical properties of copper foil, the microstructure observations of the grain size, crystal orientation, and texture are required. It is difficult to prepare samples of ultra-thin copper foils for electron back-scattered diffraction (EBSD) and transmission electron microscopy (TEM) observations in both plane and cross-section views; that is, it is not easy to polish the 8 μm thick copper foil plane without tearing. Besides, hot mounting is not suitable because copper is easily deformed by heat and pressure, and cold mounting using epoxy is not suitable due to the charging in observations using electrons. As EBSD provides crystal orientation information by identifying the Kikuchi patterns of back-scattered electrons, microstructural analysis tends to be accurate only when the polished surface is fairly uniform and flat. (Humphreys 2004; Nolze 2007; Kang and Kim 2010; Schwartz et al. 2009). The sample preparations for EBSD observations of thin foils include mechanical polishing, chemical etching and ion milling. (Randle and Engler 2010) In this study, we presents a novel specimen preparation method using only mechanical polishing for plane-view EBSD observation of 8 μm thick copper foil. Cleanup process to correct the errors that may occur during EBSD measurements was adapted and its microstructure was analyzed with consideration of TEM observations.

## Materials and methods

**Fig. 1:**
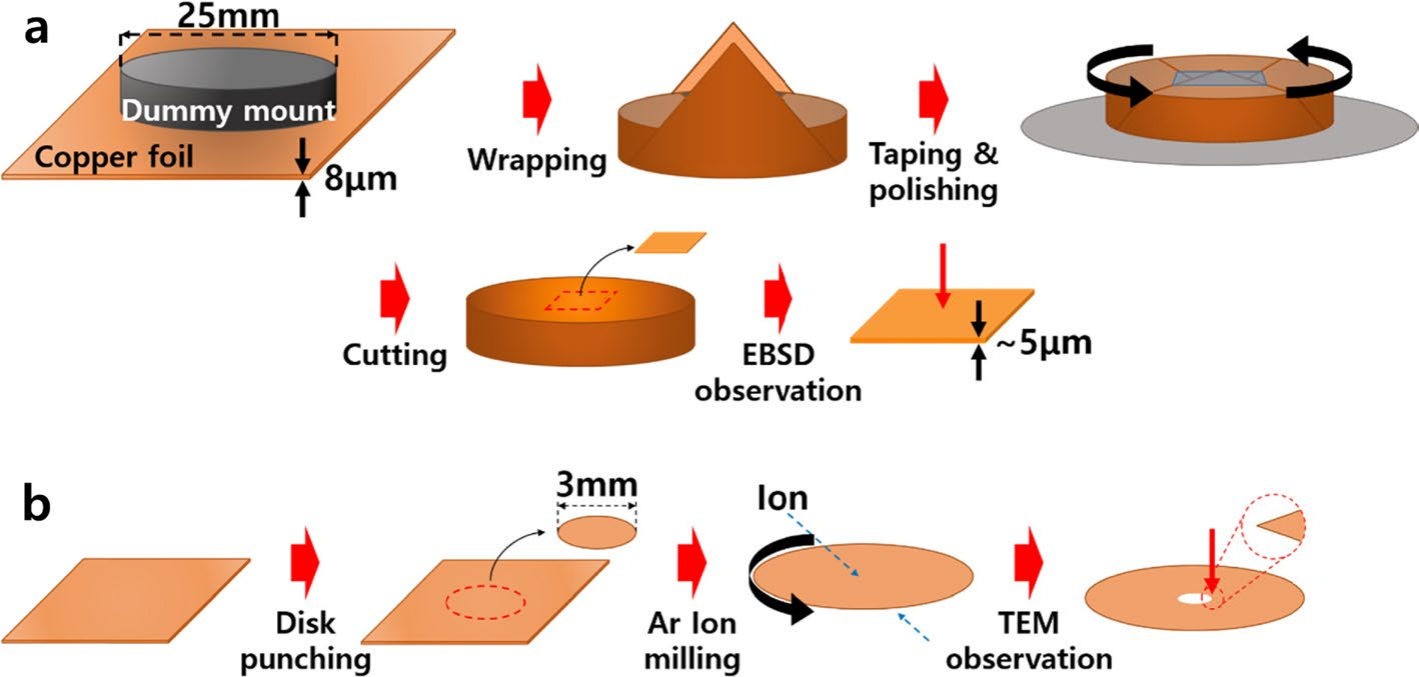
Schematic diagram of copper foil preparation for **a** EBSD and **b** TEM in plane view

The 8 μm thick copper foil was deposited on a titanium rotating drum from a copper electrolyte where it was connected to a DC voltage source. A dummy carbon mount (diameter of 25 mm) was wrapped with copper foil in order to polish the plane of copper foil, as shown in Fig. 1a. The dummy mount wrapped with copper foil was set into the groove (diameter of 26 mm) of auto polisher (MetPrep 3, Allied High Tech Products Inc.), which allows us to minimize the surface damage and tearing of copper foil. The copper foil was consecutively polished with SiC abrasive paper (#2400), 3 μm diamond suspension and 0.04 μm colloidal silica suspension. Detailed procedures of mechanical polishing conditions were shown in Table 1. Finally, the desired area was cut after grinding the copper foil to about 5 μm thickness and inserted into the specimen holder of the SEM. In this method, we could obtain not only fairly uniform and flat surface of thin copper foil but also high electrical conductivity during EBSD observations.

**Table 1:** Procedures of mechanical polishing for copper foil preparation

	**Surface**	**Abrasive**	**Time** **(min)**	**Platen Speed** **(RPM), mode**	**Sample Speed** **(RPM)**	**Remarks**
1	#2400	Silicon Carbide	5	300, comp	150	Water spray
2	Gold label	3 μm Polycrystalline Diamond suspension	5	150, comp	150	Abrasive spray per 10 s
3	Final A	0.04 μm Colloidal Silica	20	150, comp	150	Abrasive spray per 6 s Water cleaning for 60 s before finish

To observe the grain size and crystal orientation distribution of copper foil, mapping was carried out via an EBSD camera (DigiView 4, EDAX) mounted on the scanning electron microscope (SEM, Quanta 200F, FEI). The step size was taken at 50 nm (one pixel), which was typically set to about 1/10 of the average grain size (EDAX Insight 2018). After the measurements, the EBSD patterns were analyzed in orientation imaging microscopy (OIM) software (OIM Analysis v8). The accuracy of measured data is expressed by confidence index (CI) that is calculated during automated indexing of the diffraction pattern (Wright and Adams 1992). For any given Kikuchi diffraction pattern, several possible orientations may be found which satisfy the diffraction conditions. The OIM software ranks these orientations using a voting scheme. The CI is given as (*V*_1_ − *V*_2_)/*V*_ideal_, where *V*_1_ and *V*_2_ are the number of votes for the first and second solutions and *V*_ideal_ is the total possible number of votes from the detected diffraction bands. The CI value lies in the range from 0 to 1. Afterward, the cleaning up in OIM, a post-processing method that corrects inaccurate data that may have occurred during mapping, was performed in two different ways of CI standardization and grain dilation. CI standardization upgrades the low-score CI to the higher CI of a neighboring point with the same orientation, and grain dilation reconstructs grains in same direction based on set values (minimum pixel of grain size, minimum CI and tolerance angles) (Wright et al. 2015).

For TEM observation, the copper foil specimen punched 3 mm in diameter was prepared by Ar ion milling using a precision ion polisher system (PIPS, Gatan 691, Gatan Inc.) (Fig. 1b). The hole was made after sputtering for 60 min at an incident angle of 4° and consecutively 30 min at 2°, where the accelerated voltage was 4 kV. To reveal the detailed grain boundaries, the specimen was observed using TEM(CM200, Philips) and microstructure was discussed compared with EBSD observations.

## Results and discussion

**Fig. 2:**
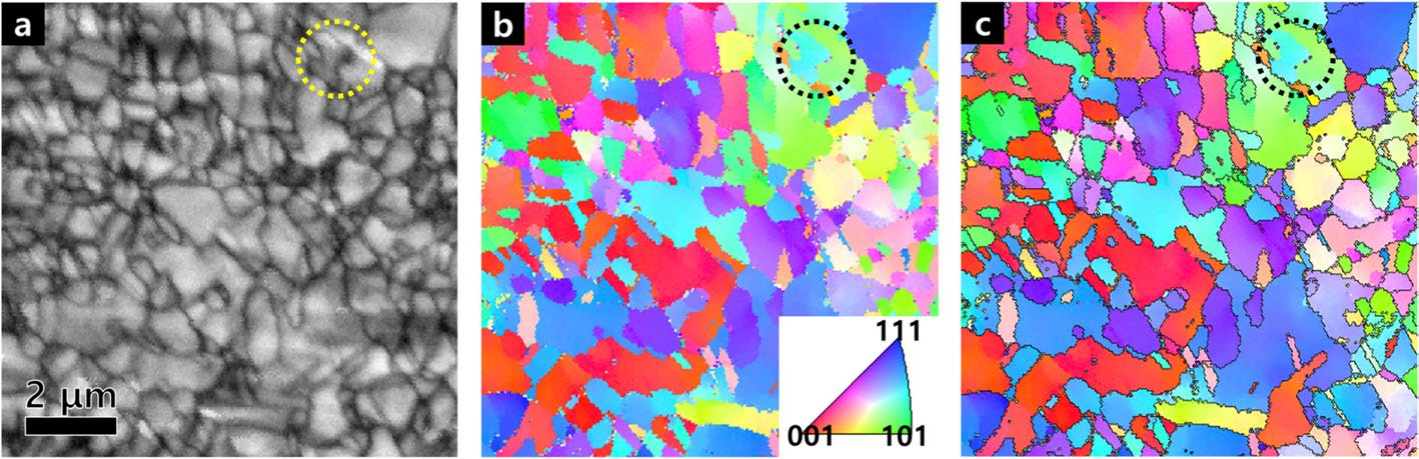
**a** Image Quality map and **b**, **c** EBSD IPF map without boundary, with boundary (rotation angle ≥ 15°) of copper foil in plane view

Figure 2 shows the image quality (IQ) and inverse pole figure (IPF) maps in plane view of copper foil by EBSD. In IQ map of Fig. 2a, constructed from electron backscatter diffraction data, provides useful visualizations of microstructure. (Wright and Nowell 2006) Grain boundaries can be clearly seen in Fig. 2a, where the grain size was measured to be approximately 365 nm. The IPF map without the grain boundaries is shown in Fig. 2b, while the boundary of rotation angle greater than 15° is depicted as black lines in Fig. 2c. Grains with random crystallographic orientations without texture can be seen in IPF maps. We observed some areas with a diameter of approximately 0.1 μm or less as indicated by black dotted circle in Fig. 2b, even though they were not clearly seen in IQ map of Fig. 2a. It is noted that there observed serrated grain boundaries in Fig. 2c.

**Fig. 3:**
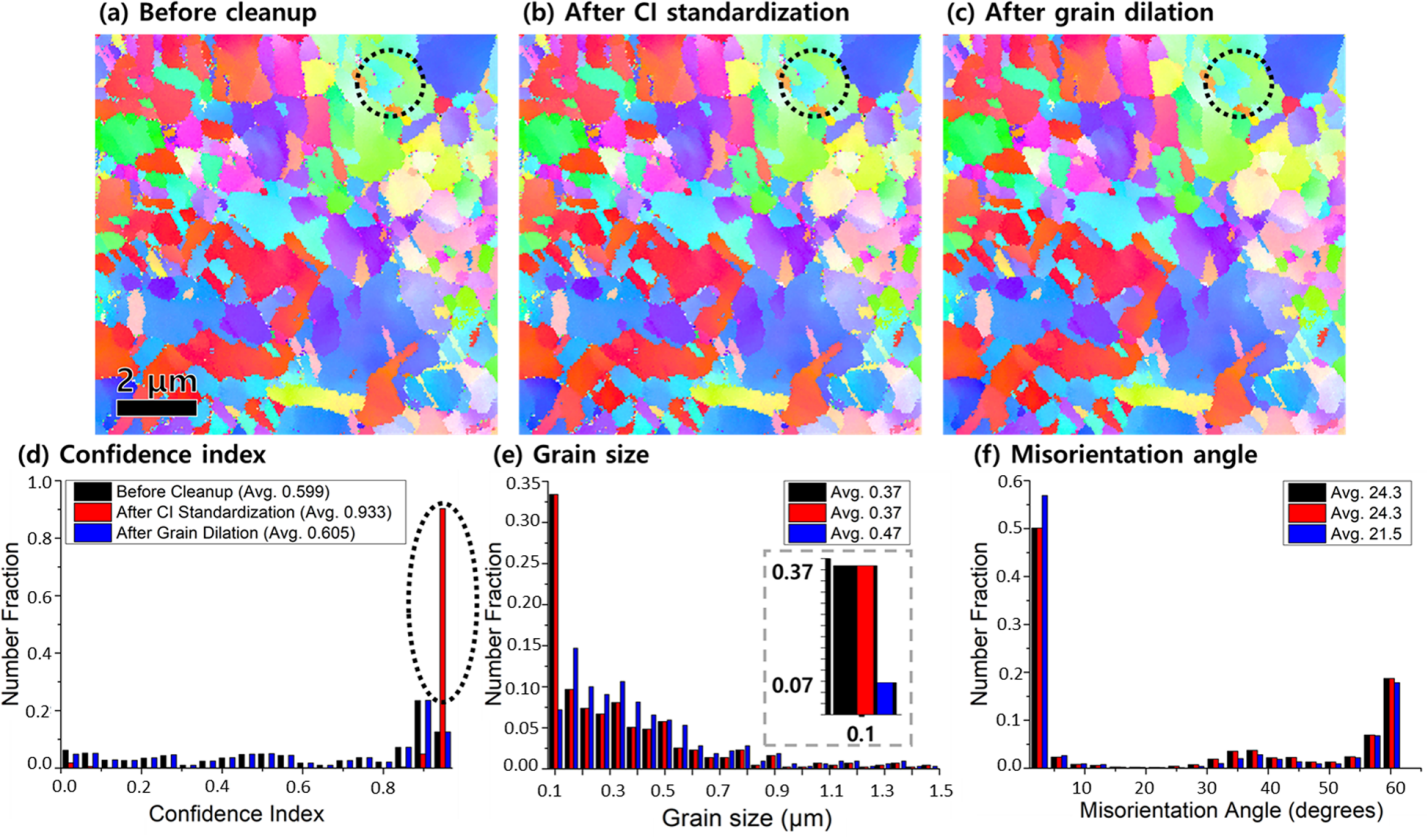
EBSD IPF map **a** before cleanup, **b** after grain CI standardization, and **c** after grain dilation of copper foil in plane view. **d** Confidence Index, **e** grain size, and **f** misorientation angle graph of **a**, **b**, and **c**

We show the IPF maps before and after two cleanup post-processing in Fig. 3. CI standardization of Fig. 3b was set to tolerance angle 5° and 5 pixels for minimum grain size, and minimum CI for grain dilation in Fig. 3c was set to 0.2 with same conditions 5° and 5 pixels of Fig. 3b. In Fig. 3b, the IPF map after CI standardization was almost identical to that before cleanup of Fig. 3a since it could not modify the orientation of the crystals but only increased the CI from 0.599 to 0.933, as marked with black dotted ellipse shown in Fig. 3d. Interestingly, after grain dilation (Fig. 3c), small areas in whole field of view disappeared as indicated by black dotted circles in Fig. 3a and c, in which the CI almost did not change, 0.599 and 0.605, respectively. We note that in the inset of Fig. 3e, the fraction of grains with diameter 0.1 μm have decreased dramatically from 33 to 7% after grain dilation cleanup and thereby the average grain size increased from 0.37 μm to 0.47 μm shown in Fig. 3e. In Fig. 3f, the fraction of high angle grains below 5° after grain dilation has been slightly increased from 50 to 57%, attributing to the disappearance of grains (rotation angle > 15°) below 5 pixels, as seen in Figs. 2c and 3c. However, serrated grain boundaries still exist after cleanup processes in Fig. 3a-c.

**Fig. 4:**
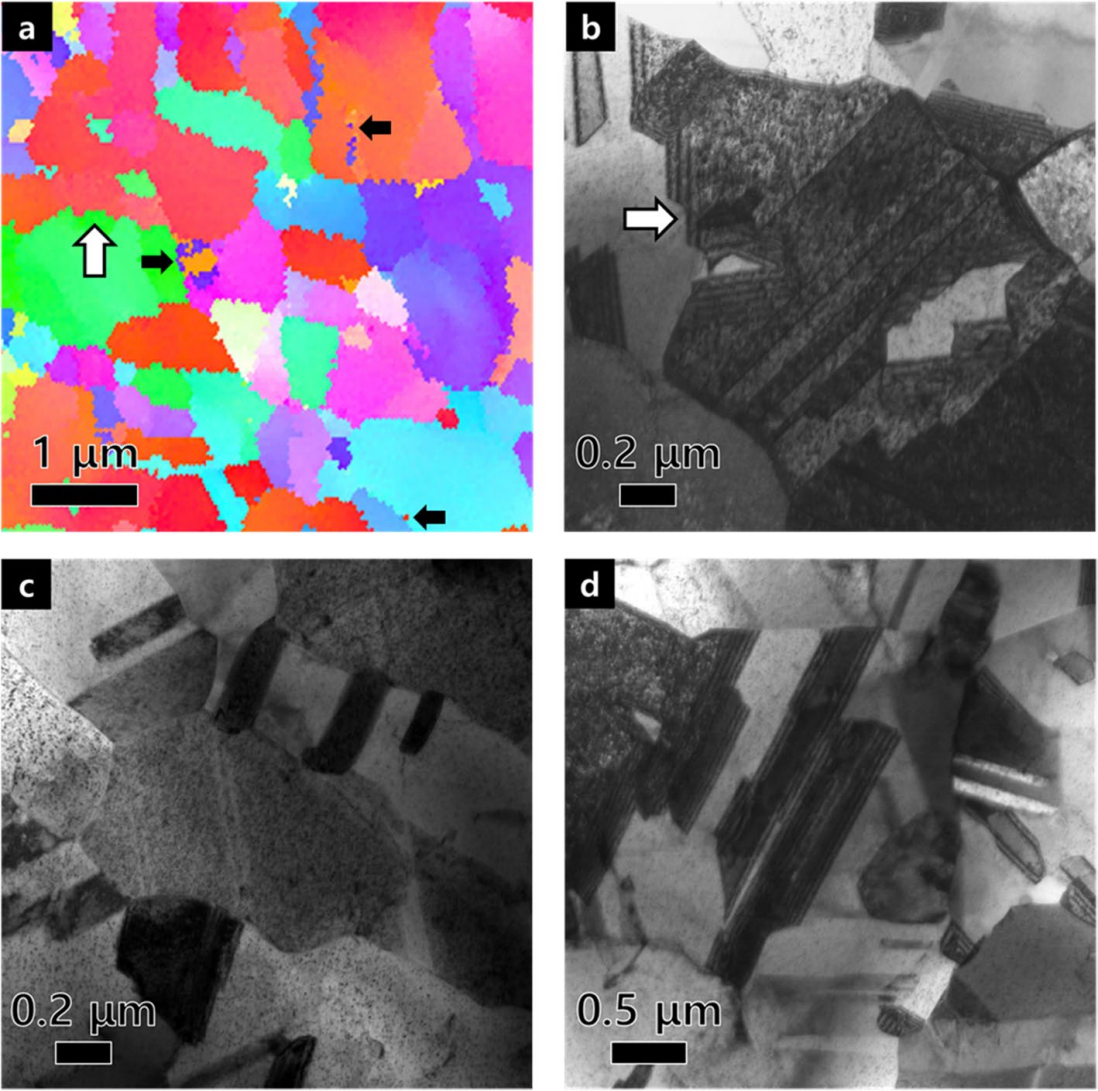
**a** EBSD IPF map after grain dilation. **b**-**d** TEM image of copper foil in plane view

We emphasize whether the images (about 0.1 μm) and serrated grain boundaries (one pitch: about 0.2 μm) revealed in EBSD IPF maps are real or artifacts. In enlarged IPF map of Fig. 4a, serrated grain boundaries can be seen clearly as indicated by white arrows in both Fig. 4a and b. A few grains with a diameter of about 0.1 μm were still attached to the grain boundaries as marked with small black arrows in Fig. 4a, even though many grains were disappeared after grain dilation cleanup in Fig. 4a. Considering whether these microstructures are real or not, IPF maps were compared with TEM images. TEM images in Fig. 4b, c and d exhibit that many grains with a diameter of about 0.3 μm exist in the copper foil plane. After the grain dilation, the number fraction less than 0.1 μm in diameter was decreased, just as in the TEM images, there are few grains with a diameter below 0.1 μm and they are lied next to the boundaries. Serrations which were observed in the boundaries on the TEM image, have pitch of about 0.2 μm similar to the IPF maps. Considering these results, we conclude that in EBSD maps, it is necessary to remove several points less than 0.1 μm (2 pixels) during the cleanup process in order not to distort the grain boundary serration. Therefore, based on our observations, the method of ‘Grain dilation’ is suitable for observing real microstructures of copper foil in plane-view using EBSD.

## Conclusions

Through the EBSD and TEM analyses, the microstructure of 8 μm thick electrolytic copper foil in plane-view was observed. Here the specimen was prepared by using only mechanical polishing, showing the advantage of being relatively simple and inexpensive rather than the focused ion beam method (FIB). In EBSD IPF maps, the cleanup process may be not only a way to improve the accuracy of the results by modifying the data initially obtained, but also one to arbitrarily distort the data. In this study, two cleanup processes (grain CI standardization and grain dilation) were then performed, which resulted in a higher CI of the map or the disappearance of points less than 0.1 μm in diameter, respectively. In EBSD IPF maps, many grains with diameter of 0.1 μm (2 pixels) were observed after grain CI standardization, while after grain dilation those (about 0.1 μm) were disappeared. From TEM observations, the grain size was verified to determine whether the artifacts caused by the cleanup or real grains contained in the copper film surface. In TEM, the small grains were measured to be about 0.3 μm in diameter and the grain boundaries serrations with one pitch of about 0.2 μm (4 pixels) were observed. On the other hand, the serration of the grain boundaries was also observed in EBSD map, showing similar pitch observed in TEM images. We consider that the method of ‘Grain dilation’ is suitable for observing real microstructures of copper foil in plane-view. These methods are expected to allow simple observation and analysis of microstructure in the plane-view of various materials with ultra-thin thickness.

## Data Availability

Please contact the corresponding author on reasonable request.

## Abbreviations

EBSD: Electron backscatter diffraction
TEM: Transmission electron microscopy
SEM: Scanning electron microscope
OIM: Orientation imaging microscopy
CI: Confidence index
PIPS: Precision ion polisher system
IQ: Image quality
IPF: Inverse pole figure

## References

[CR1] A. Ibanez, E. Fatas, Mechanical and structural properties of electrodeposited copper and their relation with the electrodeposition parameters, Surf. Coat. Technol. b1), 7–16 (2005)

[CR2] Schwartz AJ, Kumar M, Adams BL (2009). Electron backscatter diffraction in materials science.

[CR3] Insight EDAX (2018). Step Size Selection for EBSD Mapping.

[CR4] Humphreys FJ (2004). Characterisation of fine-scale microstructures by electron backscatter diffraction (EBSD). Scr. Mater..

[CR5] Nolze G (2007). Image distortions in SEM and their influences on EBSD measurements. Ultramicroscopy.

[CR6] Kang JH, Kim SH (2010). Sample preparation for EBSD analysis: Tips for metals with delicate surfaces. Korean J. Met. Mater..

[CR7] Park JK (2012). Principle and applications of lithium secondarybatteries.

[CR8] Pender JP, Jha G, Youn DH (2020). Electrode degradation in lithium-ion batteries. ACS Nano.

[CR9] Kepler KD, Vaughey JT, Thackeray MM (1999). Copper-tin anodes for rechargeable lithium batteries: an example of the matrix effect in an intermetallic system. J. Power Sources.

[CR10] Nowell MM, Witt RA, True B (2005). EBSD sample preparation: Techniques, Tips, and Tricks. Microsc. Microanal..

[CR11] Obrovac MN, Checrier VL (2014). Alloy negative electrodes for Li-ion batteries. Chem. Rev..

[CR12] R. Borah, F.R. Hughson, J. Johnston, et al., On battery materials and methods, Mater. Today Adv., 6, Article 1000046 (2020)

[CR13] Kim SH, Rhyim Y (2016). Serial sectioning and reconstruction techniques for three-dimensional microscopy of metallic materials. Korean J. Met. Mater..

[CR14] Wright SI, Adams BL (1992). Automatic analysis of electron backscatter diffraction patterns. Metall. Trans. A.

[CR15] Wright SI, Nowell MM (2006). EBSD image quality mapping. Microsc. Microanal..

[CR16] Wright SI, Nowell MM, Lindeman SP (2015). Introduction and comparison of new EBSD post-processing methodologies. Ultramicroscopy.

[CR17] Courtney TH (2000). Mechanical behavior of materials.

[CR18] Hatano T, Kurosawa Y, Miyake J (2000). Effect of material processing on fatigue of FPC rolled copper foil. J. Elec. Mater..

[CR19] Woo TG, Park IS, Seol KW (2013). Effect of additives on the elongation and surface properties of copper foils. Elec. Mater. Lett..

[CR20] Woo TG (2016). The effects of Bis(3-Sulfo-Propyl)di-Sulfide (SPS) additives on the surface morphology and mechanical properties of electrolytic copper foil. Korean J. Met. Mater..

[CR21] Randle V, Engler O (2010). Introduction to texture analysis: Macrotexture, microtexture, and orientation mapping.

